# Adult *Hyalomma marginatum* tick positive for *Rickettsia aeschlimannii* in Austria, October 2018

**DOI:** 10.2807/1560-7917.ES.2018.23.48.1800595

**Published:** 2018-11-29

**Authors:** Georg Gerhard Duscher, Adnan Hodžić, Peter Hufnagl, Walpurga Wille-Piazzai, Anna-Magarita Schötta, Mateusz Andrzej Markowicz, Agustín Estrada-Peña, Gerold Stanek, Franz Allerberger

**Affiliations:** 1 Institute of Parasitology, University of Veterinary Medicine Vienna, Austria; 2 Institute for Medical Microbiology and Hygiene, Austrian Agency for Health and Food Safety Vienna, Austria; 3 Institute for Hygiene and Applied Immunology, Medical University of Vienna, Austria; 4 Department of Animal Pathology, Faculty of Veterinary Medicine, Zaragoza, Spain

**Keywords:** *Hyalomma* spp., *Rickettsia aeschlimannii*, migratory birds

## Abstract

We report on a non-indigenous adult *Hyalomma marginatum* tick in Austria carrying the human pathogenic *Rickettsia aeschlimannii*; presumably introduced as a nymph via migratory birds and completed the moulting within the same year. It was negative for Crimean-Congo haemorrhagic fever virus, but the finding of *R. aeschlimannii* represents a potential threat for humans due to its zoonotic character. Awareness of invasive tick species and carried pathogens should be improved in central and northern Europe.

*Hyalomma marginatum* are ticks commonly found in the Mediterranean basin, Middle East and North Africa infesting humans and animals during the prevailing tick season [1-3]. It is a known vector for several viruses such as Crimean-Congo hemorrhagic fever (CCHF), Dhori, Thogoto, West Nile, but also bacteria like *Rickettsia aeschlimannii* [2], which is known to be a human pathogen [4]. Here, we present the finding of R*. aeschlimannii* in north Austria in a living adult *H. marginatum* tick collected from a horse and discuss potential public health implications.

On 2 October 2018 a male *Hyalomma marginatum* tick was removed from the hind leg of a healthy 10-year-old female Haflinger horse. The stable was located in Melk district in the state of Lower Austria (Figure) and neither the horse nor the stable owner (who removed the tick) had travelled recently. After morphological determination of the tick species at the Institute of Parasitology, University of Veterinary Medicine Vienna [5], as well as molecular confirmation of the tick species [6], the tick was checked for several pathogens such as Crimean-Congo haemorrhagic fever virus (CCHFv), *Rickettsia* species and *Babesia* species. The tick was identified as *H. marginatum* and found to be positive for *Rickettsia aeschlimannii*; no other pathogens were detected. 

**Figure:**
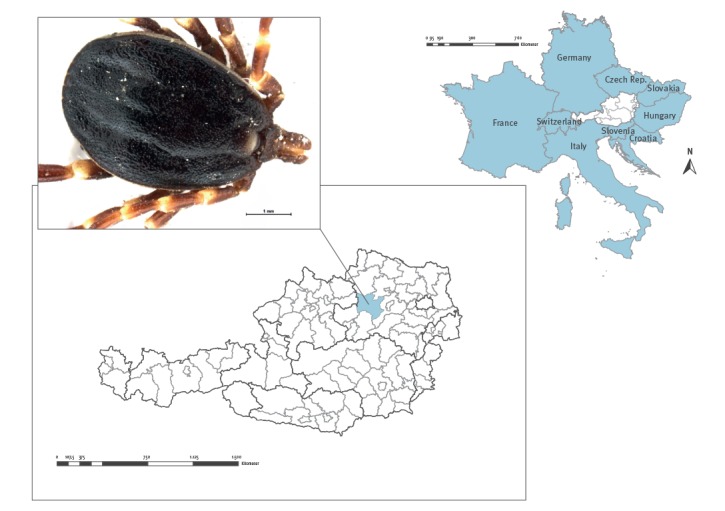
Location of the adult *Hyalomma marginatum* tick, Lower Austria, October 2018

## Investigations

DNA and RNA were extracted according to the manufacturer’s instructions with a High Pure Viral Nucleic Isolation Kit (Roche Diagnostics GmbH, Mannheim, Germany). PCRs were performed using commercial kits RealStar CCHFV RTPCR Kit 1.0 (Altona Diagnostics, Hamburg, Germany) for reverse transcription-polymerase chain reaction, or protocols targeting ITS (internal transcribed spacer) of *Rickettsia* sp. and 18S rRNA of Piroplasmidae previously published and summarised elsewhere [7]. The positive amplicon, obtained in the *Rickettsia*-specific PCR, was sent for sequencing (Microsynth, Austria) and a 344 bp fragment yielded 100% identity to *R. aeschlimannii* (GenBank AY125016). By using an additional PCR assay that amplifies a 632 bp fragment of *ompA* gene of spotted fever group (SFG) Rickettsiae [8], and a 401 bp fragment of the gltA gene [9] of *Rickettsia* spp. confirmed this finding and yielded 100% identity to the isolate HM050286 for *ompA* and 100% identity to multiple *R. aeschlimannii* strains.

## Discussion

In Europe, *Hyalomma* spp. (e.g. *H. marginatum* complex) are endemic in southern and eastern countries, including several countries in the Mediterranean [10], they were also detected in birds in countries where these ticks are not autochthonous e.g. the Czech Republic, Denmark, Finland, Germany, Norway, Poland, United Kingdom (UK), Slovak Republic and Sweden [10-12]. *Hyalomma* spp. are passively transported by migratory birds from Africa and southern Europe en route to their breeding locations in the northern hemisphere. Birds can carry the larvae and nymphs for long distances since the feeding period of these ticks may last up to 26 days [10,12]. Presumably favoured by warm weather conditions during summer and autumn, the dropped engorged nymphs can moult to adults and find a new host within the same year, even in areas considered as environmentally unsuitable for their survival [11,13]. This is why in some northern areas e.g. Germany and the Netherlands [2,13,14] adult ticks have been found on occasion, but they have not been able to establish permanent populations [3,13].

*R.* *aeschlimannii* belongs to the spotted fever group Rickettsiae [15]. In humans it causes similar symptoms to the Mediterranean spotted fever, also known as Boutonneuse fever caused by *Rickettsia conorii* and may be associated with liver dysfunction [16]. *R.* *aeschlimannii* was previously found in adult *Dermacentor*–like ticks collected from birds in Saxony-Anhalt, Germany [17], which were molecularly determined as *H. marginatum*. Here we described occurrence of *R. aeschlimannii* in infected adult *Hyalomma* spp. in another non-endemic country. It is possible that the bacteria was acquired during the blood meal from the host, however, this is unlikely as this particular pathogen was not found in any other hosts in the same geographical area.

*Hyalomma* ticks are also a vector for CCHFv. Cases of CCHF in Europe were historically described in Southeastern Europe [18]. Recent detections for the first time in humans in Spain, indicate a change of pathogen occurrence and distribution, also in this case with a potential for migratory birds to act as carriers [19]. The tick investigated here was negative for CCHFv, still the finding of *R. aeschlimannii* is of great interest, given its ability to infect humans. *R. aeschlimannii* was found in 48 of 137 ticks taken from migratory birds in Italy during 2010 and 2011 [20]; this, and our more recent finding, may indicate that with favourable weather conditions there could be more imported, moulted and attached adult *H. marginatum* found carrying *R. aeschlimannii* in Central and Northern Europe.

Potential migratory bird-linked importation of *Hyalomma* spp. to northern European countries should be taken into account in the near future. Public health systems should prepare themselves in terms of detection of ticks, diagnostic and monitoring of pathogens transmitted by these, as well as respective treatment and control measures. On behalf of the Austrian ministry of health, the Austrian Agency for Health and Food Safety Vienna (AGES), in close cooperation with the Medical University Vienna and the University of Veterinary Medicine Vienna, has set up a website [21] to inform the population about potentially emerging threats associated with ticks.
